# Shift of radiotherapy use during the first wave of the COVID-19 pandemic? An analysis of German inpatient data

**DOI:** 10.1007/s00066-021-01883-1

**Published:** 2022-01-07

**Authors:** Daniel Medenwald, Thomas Brunner, Hans Christiansen, Ulrich Kisser, Sina Mansoorian, Dirk Vordermark, Hans-Ulrich Prokosch, Susanne A. Seuchter, Lorenz A. Kapsner, our MII research group, Julien Balig, Jonas Bienzeisler, Daniel Buergy, Patrick Fischer, Jonas Fortmann, Timo Fuchs, Thomas Ganslandt, Matthias Gietzelt, Christian Haverkamp, Kurt Marquardt, Dennis Kadioglu, Irina Lutz, Gerhard Mayer, Achim Michel-Backofen, Harald Renz, Christian Seidemann, Holger Stenzhorn, Ana Stolnicu, Holger Storf, Gaetan Kamdje Wabo, Jochen Zohner

**Affiliations:** 1 Department of Radiation Oncology, University Hospital Halle (Saale), Ernst-Grube-Straße 40, 06120 Halle (Saale), Germany; 2 Department of Radiation Oncology, University Medical Center Magdeburg, Magdeburg, Germany; 3 Department of Radiation Oncology, Hannover Medical School, Hannover, Germany; 4 Department of Otorhinolaryngology, Head and Neck Surgery, University Clinic Halle, Halle, Germany; 5 Department of Radiation Oncology, Universitätsklinikum Erlangen, Friedrich-Alexander-Universität Erlangen-Nürnberg (FAU), Erlangen, Germany; 6 Chair of Medical Informatics, Friedrich-Alexander-Universität Erlangen-Nürnberg (FAU), Erlangen, Germany; 7 Medical Center for Information and Communication Technology, Universitätsklinikum Erlangen, Erlangen, Germany; 8 Department of Radiology, Universitätsklinikum Erlangen, Friedrich-Alexander-Universität Erlangen-Nürnberg (FAU), Erlangen, Germany

**Keywords:** Covid-19 pandemic, Lockdown, Radiotherapy, Germany, Admissions

## Abstract

**Objective:**

To assess the change in inpatient radiotherapy related to COVID-19 lockdown measures during the first wave of the pandemic in 2020.

**Methods:**

We included cases hospitalized between January 1 and August 31, 2018–2020, with a primary ICD-10 diagnosis of C00–C13, C32 (head and neck cancer, HNC) and C53 (cervical cancer, CC). Data collection was conducted within the Medical Informatics Initiative. Outcomes were fractions and admissions. Controlling for decreasing hospital admissions during holidays, calendar weeks of 2018/2019 were aligned to Easter 2020. A lockdown period (LP; 16/03/2020–02/08/2020) and a return-to-normal period (RNP; 04/05/2020–02/08/2020) were defined. The study sample comprised a control (admission 2018/19) and study cohort (admission 2020). We computed weekly incidence and IR ratios from generalized linear mixed models.

**Results:**

We included 9365 (CC: 2040, HNC: 7325) inpatient hospital admissions from 14 German university hospitals. For CC, fractions decreased by 19.97% in 2020 compared to 2018/19 in the LP. In the RNP the reduction was 28.57% (*p* < 0.001 for both periods). LP fractions for HNC increased by 10.38% (RNP: 9.27%; *p* < 0.001 for both periods). Admissions for CC decreased in both periods (LP: 10.2%, RNP: 22.14%), whereas for HNC, admissions increased (LP: 2.25%, RNP: 1.96%) in 2020. Within LP, for CC, radiotherapy admissions without brachytherapy were reduced by 23.92%, whereas surgery-related admissions increased by 20.48%. For HNC, admissions with radiotherapy increased by 13.84%, while surgery-related admissions decreased by 11.28% in the same period.

**Conclusion:**

Related to the COVID-19 lockdown in an inpatient setting, radiotherapy for HNC treatment became a more frequently applied modality, while admissions of CC cases decreased.

**Supplementary Information:**

The online version of this article (10.1007/s00066-021-01883-1) contains supplementary material, which is available to authorized users.

## Introduction

The first wave of the COVID-19 pandemic, spreading globally in the spring of 2020, affected all areas of the health sector. While disciplines directly managing patients with proven SARS-CoV‑2 infection, such as pneumonology or intensive care, were hardest hit initially, the multidisciplinary management of cancer patients was also immediately affected.

In particular, the risk of limited availability of intensive care units led to a call from the German Ministry of Health to delay all non-urgent surgeries in all hospitals to free up intensive care capacity on March 12, 2020. Whereas cancer treatment is not considered elective or non-urgent, national and international expert groups devised recommendations on the interdisciplinary management of cancer patients under conditions of limited resources [1].

A common pattern of these recommendations was the consideration to avoid major surgery in cancer patients who would be expected to require postoperative intensive care and instead prefer non-surgical treatments including radiotherapy [2]. Leading German radiation oncology institutions published recommendations on the management of radiation oncology departments during the COVID-19 pandemic, including strategies to handle shortages of staff and the continuation of radiotherapy in patients with suspected or proven SARS-CoV‑2 infection [3]. In addition, these recommendations mention hypofractionated schedules that would result in fewer administered fractions during the pandemic [4].

One comprehensive study from Great Britain revealed a considerable decrease in the number of radiotherapy courses across all entities in 2020 when compared to 2019 [5]. It is unclear to what extent the use of radiotherapy in cancer treatment was affected during the first wave of the COVID-19 pandemic in Germany. Recently published survey results from German comprehensive cancer centers (CCCs) indicated that radiotherapy availability was never affected during the first wave as opposed to, e.g., diagnostic imaging, systemic therapy, or cancer surgery [6]. A questionnaire survey among radiation oncology institutions in Germany, Austria, and Switzerland performed in April and May 2020 documented a decrease in the number of patients treated with radiotherapy, which was unrelated to the incidence of SARS-CoV-2-positive cases or the type of radiation oncology institution [7]. The survey respondents reported a tendency to change the fractionation mostly in palliative radiotherapy concepts and to postpone radiotherapy in curative indications.

The German Medical Informatics Initiative (MII) [8], comprising the four consortia DIFUTURE [9], HiGHmed [10], MIRACUM [11], and SMITH [12], previously established an infrastructure to support federated analyses across the participating German university hospitals [13]. Based on this infrastructure, we are now able to analyze possible changes in radiotherapeutic inpatient settings caused by the COVID pandemic.

Our analysis focuses on cervical cancer (CC) and head and neck cancer (HNC), as in both entities, primary radiochemotherapy is an alternative option to primary surgical treatment according to stage and risk factors [14–16]. In addition, as many institutions administer radiochemotherapy in an inpatient setting, both entities might well serve as surrogates to analyze COVID-19 effects in an inpatient collective. In CC, radical hysterectomy is the mainstay of curative treatment for early stages in Germany, but radiotherapy is stated as an alternative in the S3 guidelines [17]. Furthermore, in many situations of HNC, including laryngeal cancer requiring laryngectomy, radical radiotherapy or chemoradiation are organ-conserving alternatives to radical surgery [18].

Thus, additional use of radiotherapy in the management of CC or HNC could become apparent via increased inpatient delivery of radiotherapy or chemoradiation, with a simultaneous reduction in the number of cases treated with surgery. A large proportion of institutions administer radiotherapy, especially brachytherapy or chemoradiation, in an inpatient setting, which is, however, not the case for the treatment with radiotherapy alone. Based on this reasoning, we aim to assess the effect of the lockdown on fractions and admissions in an inpatient setting in radiotherapy institutions.

CC and HNC are two cancer types that are typically treated by definitive chemoradiotherapy and in which no delays of therapy can be accepted, with respect to the rapid proliferation of these tumors. Concomitant radiotherapy and chemotherapy are usually performed by the radiation oncologist. Therefore, treatment of these two diseases is less dependent on biases caused by multidisciplinary treatment compared to many other tumor entities, supporting the choice of these cancer types with relatively robust rates of incidence, diagnosis, and treatment within radiation oncology departments.

## Methods

We used claims data from 14 university hospitals to analyze the change in treatment of CC or HNC following the lockdown announcement on March 16, 2020, in Germany [19]. The study was approved by the ethics committee of the Friedrich-Alexander-University (FAU) Erlangen-Nürnberg (259_20 Bc) and approval was further obtained at the respective responsible local ethics committees as well as from their use and access committees (UACs) by each participating site.

### Cohort selection

Data from inpatients fulfilling the following criteria were included in the study:

- Eligible cases were identified by a matching principal ICD code excluding the secondary ICD codes C78 and C79 (secondary malignant). Based on their principal ICD codes, cases were grouped as *malignant neoplasm of cervix uteri* or *malignant neoplasm of head & neck*. We allocated cases to seven treatment groups in accordance with the recorded OPS codes (Table 1; see Supplementary Table S1 for further details).
- Inpatient hospital admission between January 1 and August 31 of the years 2018, 2019, and 2020.
- All cases had to be complete, i.e., a discharge date had to be present at the time of data retrieval.

**Table 1:** Treatment groups

Treatment group	Diagnosis group	Therapy category
1	Malignant neoplasm of head & neck	Surgery present
2		Radiotherapy w/o surgery
3		Radiotherapy w/o surgery, chemotherapy present
4	Malignant neoplasm of cervix uteri	Surgery present
5		Radiotherapy w/o surgery, chemotherapy present
6		Radiotherapy w/o surgery w/o brachytherapy
7		Radiotherapy w/o surgery, brachytherapy present

The seven treatment groups analyzed within this study are formed by a combination of diagnosis groups and therapy categories. A detailed listing of the respective inclusion and exclusion criteria is provided with Supplementary Table S1

### Data retrieval and data transformation

Data collection was conducted on the infrastructure built by the MIRACUM consortium and delivered to MII sites as previously published in [13]. For eligible inpatient encounters, each participating site’s research data repository was queried for the following data elements:

- Principal diagnosis (primary codes based on ICD-10-GM; www.dimdi.de).
- Related procedure codes (available as *Operationen- und Prozedurenschlüssel* [OPS]) and corresponding timestamps.
- Begin of inpatient stay (granularity of calendar days).
- End of inpatient stay (granularity of calendar days).
- Pseudonymized patient identifier (ID) and encounter ID.

The data were further transformed in order to derive the following data elements:

- Radiotherapeutic procedures (OPS codes 8‑52*) per calendar week, further stratified by the subgroups 8‑52(2,3)* [megavoltage radiation therapy] and 8‑52(4,5)* [brachytherapy], respectively.

### Outcomes

We compared the *number of radiotherapy fractions* as primary outcome and *inpatient hospital admissions *as a secondary outcome of the year 2020 to the average of the years 2018 and 2019 (using the average to avoid year-specific fluctuations). Observed events within this study are the weekly counts of those outcomes. To control for decreasing inpatient hospital admissions in connection with holiday periods, calendar weeks (cw) of 2018 and 2019 were aligned with the timing of Easter holidays in 2020 as previously described and further referred to as “adjusted weeks” [13].

Frequency counts aggregated across all participating sites were analyzed in more detail for two time periods:

A. The “lockdown period” (LP) is defined as March 16, 2020, (i.e., cw 12/adjusted week 0) to August 2, 2020, (i.e., cw 31/adjusted week 19).
B. A second period (return to normal, RNP) is defined as May 4 (i.e., cw 19/adjusted week 7) to August 2, 2020, since on April 28, 2020, the German Federal Ministry of Health announced the gradual reactivation of hospital capacity for elective surgeries from May onwards [20, 21].

The study sample was divided into two cohorts: 1) a study cohort of cases hospitalized in 2020; 2) a control cohort of cases hospitalized in 2018 and 2019.

For the primary outcome of *number of radiotherapeutic fractions, *the underlying radiotherapeutic procedure codes were grouped by cw based on the respective date. The resulting numbers reflect the procedures performed in a specific cw across all sites. In contrast, inpatient hospital admission numbers are counted for the adjusted week in which the patient was actually admitted.

These aggregated data were centrally collected with the infrastructure of the University Hospital Erlangen (UHE) as previously described [13].

### Statistical analysis

Changes between study and control cohort are reported as absolute and relative differences for the corresponding periods. Statistical analyses were performed with R version 4.0.4 (R core team, Vienna) [22].

Weekly incidence rates (IR) were calculated for each period by dividing the cumulative sum across all sites for each outcome and cohort by the number of weeks in the respective period. Generalized linear mixed models (GLMM) with Poisson distribution were implemented to calculate incidence rate ratios (IRRs) between study and control periods. For each outcome and period, a separate model was calculated with the cumulative number of weekly events for each site as the dependent variable and the cohort as the independent variable, specifying the hospital site as a random factor. We checked for over- as well as under-dispersion and found no evidence [23]. When the average of 2018 and 2019 was computed, non-integer values in the control cohort were rounded up if the ratio of the cumulative sum of events per site between study cohort and control cohort was greater than one; otherwise, they were rounded down.

## Results

A total of 9365 inpatient hospital admissions from 14 German university hospitals met the cohort selection criteria (range per site: 258–1759, Table 2). A total of 2040 inpatient hospital admissions were related to malignant neoplasm of the cervix uteri (range per site: 12–607, Table 2). We identified 7325 inpatient hospital admissions related to HNC (range per site: 204–1152, Table 2).

**Table 2:** Distribution of inpatient hospital admissions across all 14 participating sites

	Study sample	Malignant neoplasm of cervix uteri	Malignant neoplasm of head & neck
	Minimum/median/maximum (IQR)		
Total admissions per site	258/584.5/1759 (158.25)	12/124/607 (42.5)	204/494.5/1152 (131.5)
2018	95/202/573 (56.75)	6/42/205 (18.25)	74/160.5/368 (70.75)
2019	52/191.5/554 (61)	6/36.5/200 (25.25)	40/169.5/354 (64.5)
2020	80/201.5/632 (58.25)	0/41/202 (20.75)	80/158/430 (57.5)

*IQR* interquartile range

### Cervical cancer

**Fig. 1:**
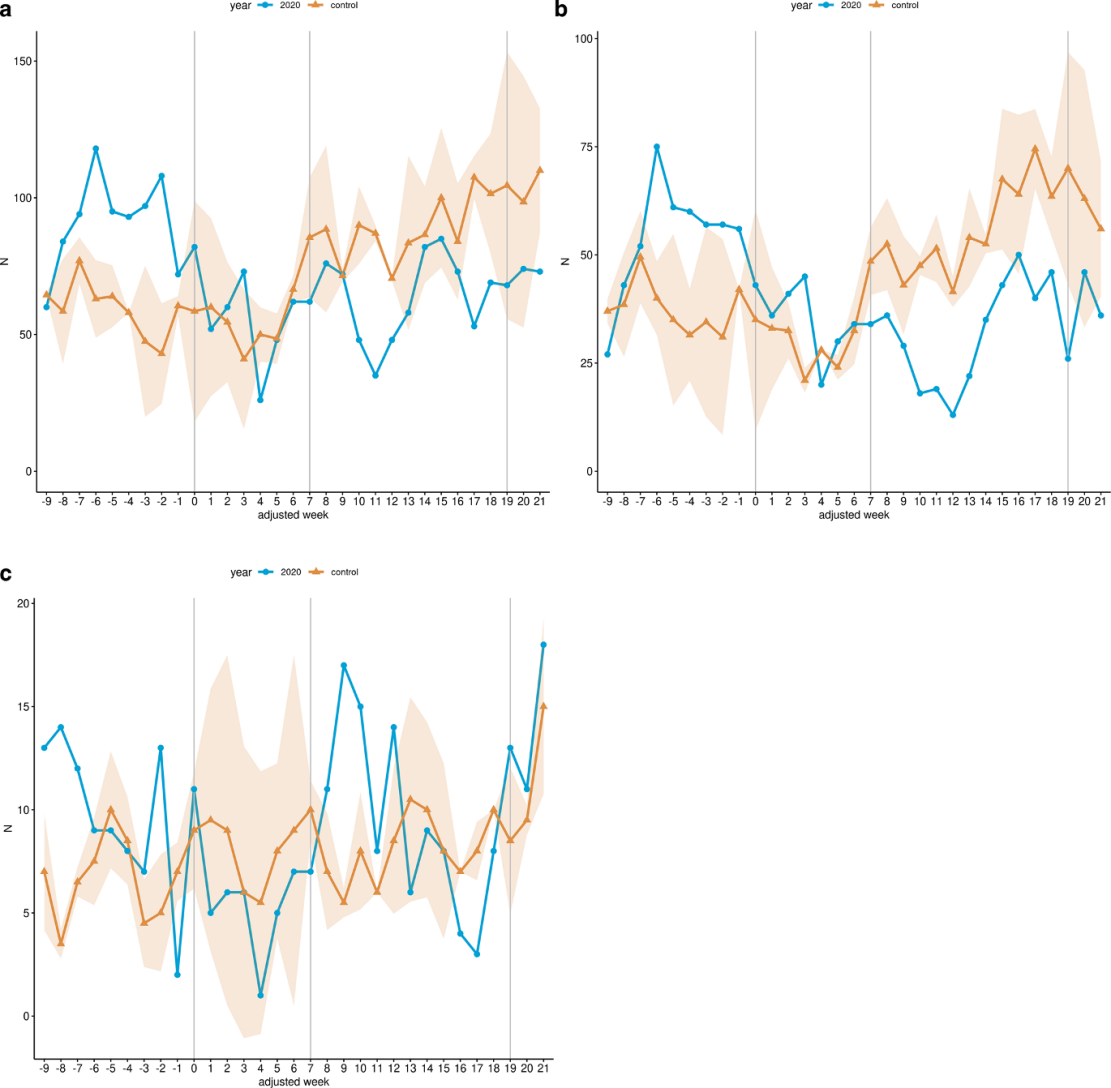
Radiotherapeutic fractions for malignant neoplasm of cervix uteri. Line charts of the cumulative weekly performed radiotherapeutic fractions related to malignant neoplasms of cervix uteri across all 14 participating sites for the study cohort (*blue*) and the control cohort (*yellow*) from January 13 (adjusted week -9) to August 16 (adjusted week 21) 2020. **a** Overall radiotherapeutic fractions (OPS 8‑52*); **b** Megavoltage radiation therapy (OPS 8‑522*, 8‑523*); **c** Brachytherapy (OPS 8-524*, 8-525*). The *shaded area* represents the standard deviation of the weekly average of the years 2018 and 2019. Additionally, the data are shown without time resolution as boxplots in supplemental Figure S1

Fig. 1 illustrates the weekly performed radiotherapeutic fractions from January 13 (adjusted week −9) to August 16 (adjusted week 21) 2020. In the LP, radiotherapeutic fractions decreased by 19.97% (1232 to 1539.5, *p* < 0.001) in the study cohort compared to the control cohort (Fig. 1a; Table 3). Megavoltage radiation therapy decreased by 29.52% (660 to 936.5, *p* < 0.001; Fig. 1b; Table 3), whereas no change was observed for brachytherapy-related fractions (164 to 163, *p* ≥ 0.05; Fig. 1c; Table 3).

**Table 3:** Weekly incidence rates of performed radiotherapeutic fractions across all 14 participating sites

	LP			RNP		
Radiotherapeutic fraction subgroup	Study cohort (2020)	Control cohort	IRR (95% CI)	Study cohort (2020)	Control cohort	IRR (95% CI)
*Malignant neoplasm of cervix uteri*						
Any radiation therapy (8-52*)	61.6	76.97	0.8 (0.74–0.86)***^,b^	63.77	89.27	0.72 (0.66–0.78)***^,b^
Megavoltage radiation therapy (8-522, 8‑523)	33	46.83	0.71 (0.64–0.78)***^,b^	31.62	56.19	0.57 (0.5–0.64)***^,b^
Brachytherapy (8-524, 8‑525)	8.2	8.15	1 (0.8–1.24)^a^	9.46	8.23	1.14 (0.88–1.47)^a^
*Malignant neoplasm of head & neck*						
Any radiation therapy (8-52*)	413.5	374.62	1.1 (1.07–1.14)***^,a^	408.92	374.23	1.09 (1.05–1.14)***^,a^
Megavoltage radiation therapy (8-522, 8‑523)	346.15	315.18	1.1 (1.06–1.14)***^,a^	351.46	311.85	1.13 (1.08–1.18)***^,a^

*LP* lockdown period, *RNP* return-to-normal period, *w/o* without, *95% CI* 95% confidence interval, *IRR* incidence rate ratio

*P*-value significance codes: *: < 0.05, **: < 0.01, ***: < 0.001

^a^Non-integer values in the control cohort were rounded up before applying the generalized linear mixed model (GLMM) in case of a ratio of study cohort to control cohort of >1

^b^Non-integer values in the control cohort were rounded down before applying the GLMM in case of a ratio of study cohort to control cohort of <1

Within the RNP, the reduction in overall radiotherapeutic fractions was 28.57% (829 to 1160.5, *p* < 0.001; Fig. 1a), whereas megavoltage radiation therapy fractions decreased by 43.74% (411 to 730.5, *p* < 0.001; Fig. 1b; Table 3) and brachytherapy fractions even increased by 14.95% (123 to 107, *p* ≥ 0.05; Fig. 1c; Table 3) in 2020 in comparison with the control cohort.

**Fig. 2:**
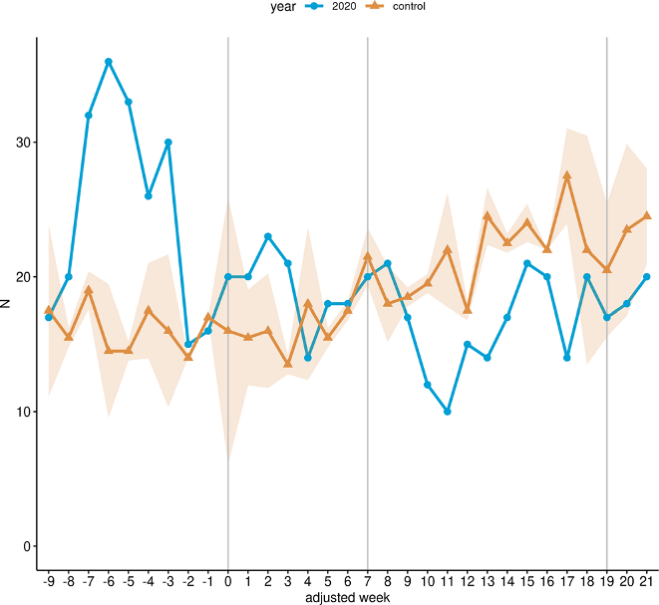
Hospital admissions for malignant neoplasm of cervix uteri. Line chart of the cumulative weekly hospital admissions related to malignant neoplasms of cervix uteri across all 14 participating sites for the study cohort (*blue*) and the control cohort (*yellow*) from January 13 (adjusted week -9) to August 16 (adjusted week 21) 2020. The *shaded area* represents the standard deviation of the weekly average of the years 2018 and 2019. Additionally, the data are shown without time resolution as a boxplot in supplemental Figure S2

**Fig. 3:**
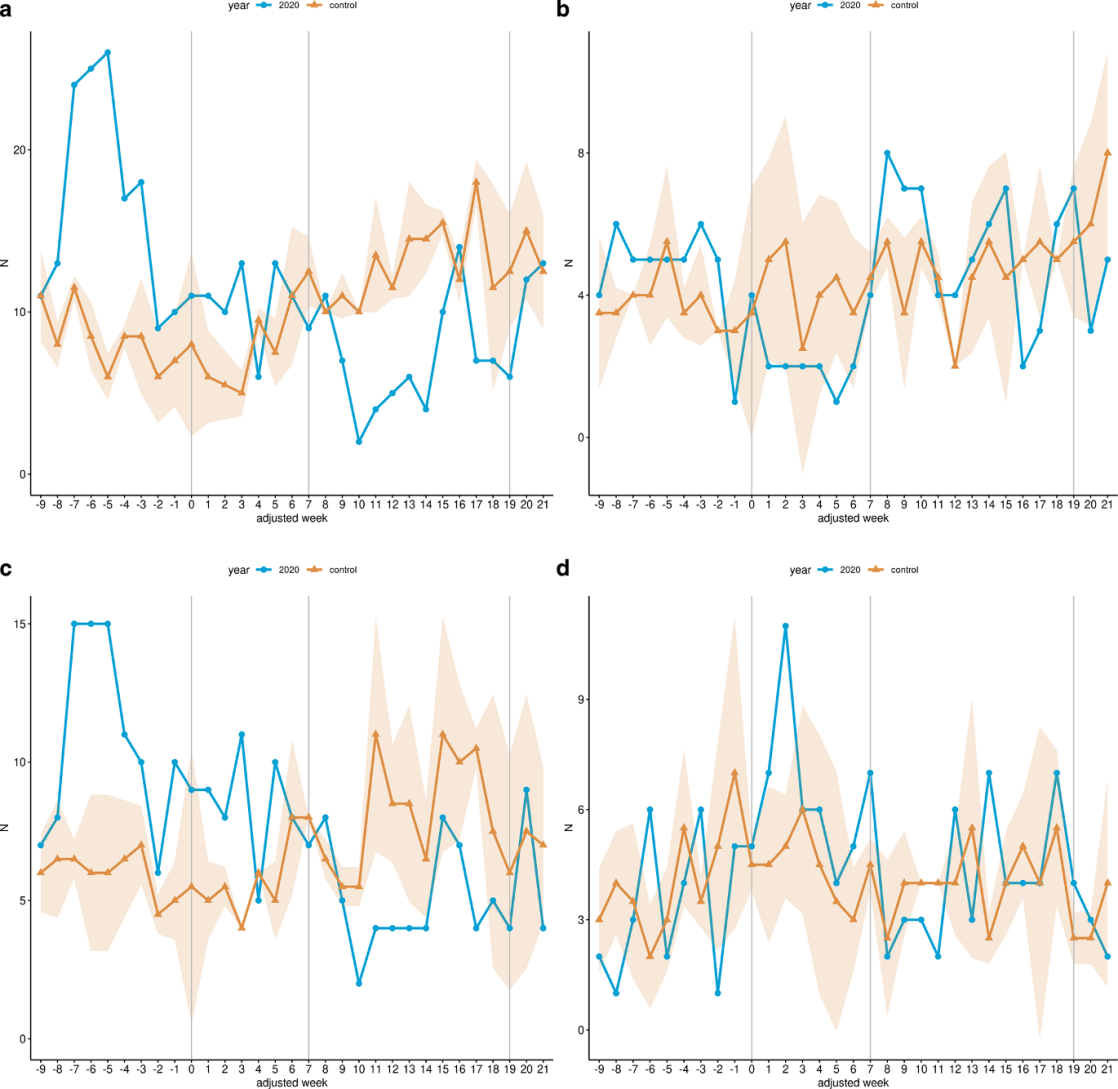
Hospital admissions for malignant neoplasm of cervix uteri stratified by treatment groups. Line charts of the cumulative weekly hospital admissions related to malignant neoplasms of cervix uteri across all 14 participating sites for the study cohort (*blue*) and the control cohort (*yellow*) from January 13 (adjusted week -9) to August 16 (adjusted week 21) 2020 stratified by therapy categories. **a** Hospital admissions with radiotherapeutic procedures, without surgery-related procedures and without brachytherapy. **b** Hospital admissions with radiotherapeutic procedures, without surgery-related procedures and presence of brachytherapy. **c** Hospital admissions with radiotherapeutic procedures, without surgery-related procedures and presence of chemotherapy. **d** Hospital admissions with presence of surgery-related procedures. The *shaded area* represents the standard deviation of the weekly average of the years 2018 and 2019. Additionally, the data are shown without time resolution as boxplots in supplemental Figure S3

Within the LP, overall hospital admissions for cervical cancer fell by 10.2% (352 to 392, *p* > 0.05; Table 4) in the study cohort compared to the control cohort, whereas a reduction of 22.14% (218 to 280, *p* < 0.01) was observed in the RNP (Fig. 2). Radiotherapy admissions without brachytherapy were reduced by 23.92% (167 to 219.5, *p* < 0.05; Fig. 3a; Table 4), whereas admissions with chemotherapy procedures were reduced by 12.5% (126 to 144, *p* ≥ 0.05; Fig. 3c), and brachytherapy-related admissions were reduced by 5.03% (85 to 89.5, *p* ≥ 0.05; Fig. 3b; Table 4) within the LP in 2020 compared with the control cohort. In contrast, admissions with surgery-related procedures increased non-significantly by 20.48% (100 to 83, *p* ≥ 0.05) in the same period (Fig. 3d; Table 4). For the RNP, radiotherapy admissions without brachytherapy decreased by 44.91% (92 to 167, *p* < 0.001; Fig. 3a; Table 4), which was also true for admission with radio- and chemotherapy (37.14%; 66 to 105, *p* < 0.01; Fig. 3c; Table 4). In contrast, radiotherapeutic admissions with brachytherapy increased by 14.75% (70 to 61; Fig. 3b; Table 4) and admissions with surgical procedures increased by 7.69% (56 to 52, *p* ≥ 0.05) in the same period compared with the control cohort (Fig. 3d; Table 4).

**Table 4:** Weekly incidence rates of inpatient hospital admissions across all 14 participating sites

	LP			RNP		
	Study cohort (2020)	Control cohort	IRR (95% CI)	Study cohort (2020)	Control cohort	IRR (95% CI)
*Malignant neoplasm of cervix uteri*						
Total admissions per week	17.6	19.6	0.9 (0.78–1.04)^b^	16.77	21.54	0.79 (0.66–0.94)**^,b^
Radiotherapy w/o surgery w/o brachytherapy	8.35	10.97	0.78 (0.64–0.95)*^,b^	7.08	12.85	0.56 (0.44–0.73)***^,b^
Radiotherapy w/o surgery, brachytherapy present	4.25	4.47	0.99 (0.74–1.33)^b^	5.38	4.69	1.11 (0.8–1.55)^a^
Radiotherapy w/o surgery, chemotherapy present	6.3	7.2	0.89 (0.7–1.13)^b^	5.08	8.08	0.64 (0.47–0.87)**^,b^
Surgery present	5	4.15	1.2 (0.9–1.61)^a^	4.31	4	1.12 (0.77–1.63)^a^
*Malignant neoplasm of head & neck*						
Total admissions per week	69.35	67.83	1.02 (0.95–1.1)^a^	68.15	66.85	1.02 (0.93–1.12)^a^
Radiotherapy w/o surgery	40.1	35.23	1.14 (1.03–1.26)*^,a^	39.31	34.23	1.15 (1.01–1.31)*^,a^
Radiotherapy w/o surgery, chemotherapy present	27.85	25.05	1.11 (0.98–1.25)^a^	27.08	24.69	1.1 (0.94–1.27)^a^
Surgery present	26.55	29.92	0.89 (0.79–1)^b^	26.31	30.35	0.87 (0.76–1.01)^b^

*LP* lockdown period, *RNP* return-to-normal period, *w/o* without, *95% CI* 95% confidence interval, *IRR* incidence rate ratio

*P*-value significance codes: *: < 0.05, **: < 0.01, ***: < 0.001

^a^Non-integer values in the control cohort were rounded up before applying the generalized linear mixed model (GLMM) in case of a ratio of study cohort to control cohort of >1

^b^Non-integer values in the control cohort were rounded down before applying the GLMM in case of a ratio of study cohort to control cohort of <1

### Head and neck cancer

**Fig. 4:**
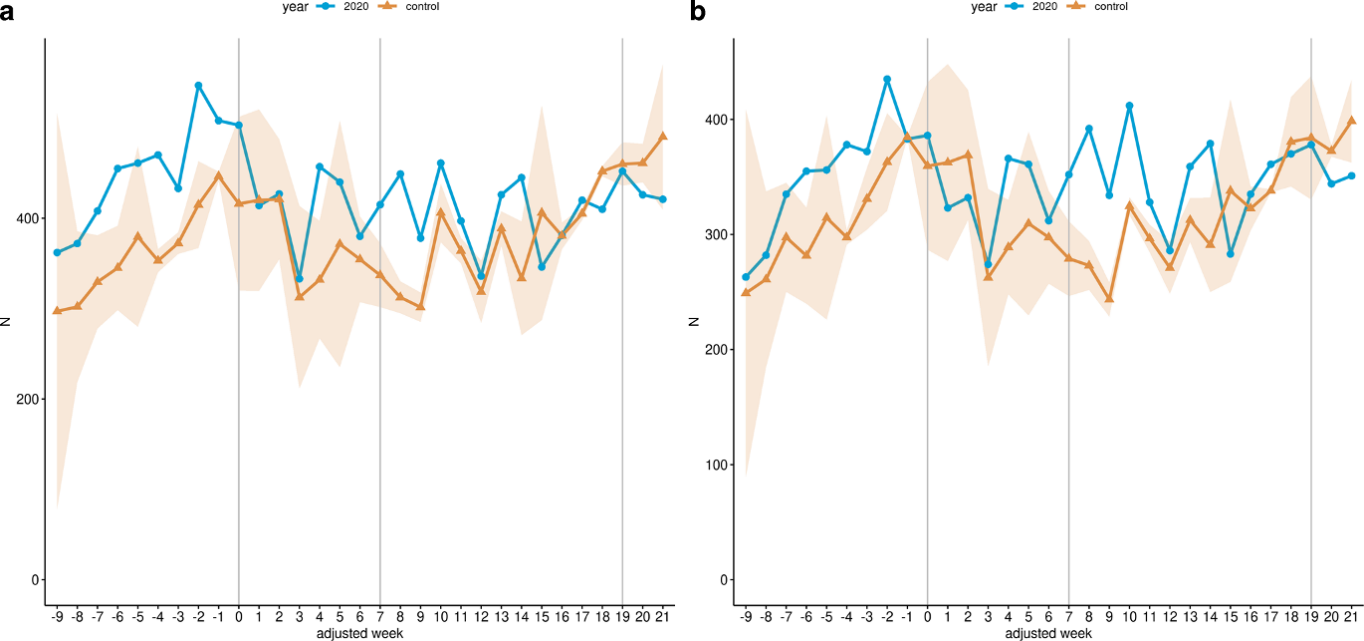
Radiotherapeutic fractions for malignant neoplasm of head & neck. Line charts of the cumulative weekly performed radiotherapeutic fractions related to malignant neoplasms of head & neck across all 14 participating sites for the study cohort (*blue*) and the control cohort (*yellow*) from January 13 (adjusted week -9) to August 16 (adjusted week 21) 2020. **a** Overall radiotherapeutic fractions (OPS 8‑52*); **b** Megavoltage radiation therapy (OPS 8‑522*, 8‑523*). The *shaded area* represents the standard deviation of the weekly average of the years 2018 and 2019. Additionally, the data are shown without time resolution as boxplots in supplemental Figure S4

**Fig. 5:**
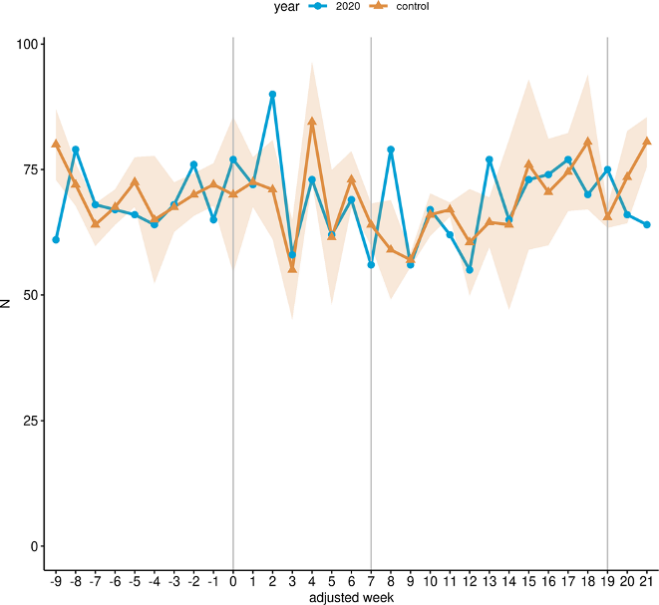
Hospital admissions for malignant neoplasm of head & neck. Line chart of the cumulative weekly hospital admissions related to malignant neoplasms of head & neck across all 14 participating sites for the study cohort (*blue*) and the control cohort (*yellow*) from January 13 (adjusted week –9) to August 16 (adjusted week 21) 2020. The *shaded area* represents the standard deviation of the weekly average of the years 2018 and 2019. Additionally, the data are shown without time resolution as a boxplot in supplemental Figure S5

Within the LP, overall radiotherapy fractions increased by 10.38% (8270 to 7492.5, *p* < 0.001) in the study cohort compared to the control cohort (Fig. 4a; Table 3). Megavoltage radiation therapy fractions increased by 9.83% (6923 to 6303.5, *p* < 0.001; Fig. 4b; Table 3). Within the RNP the increase in overall radiotherapy fractions was 9.27% (5316 to 4865, *p* < 0.001; Fig. 4a; Table 3), whereas megavoltage radiation therapy fractions rose by 12.7% (4569 to 4054, *p* < 0.001; Fig. 4b; Table 3) in the same period in 2020 in comparison with the control cohort. Within the LP, overall hospital admissions for HNC increased by 2.25% (1387 to 1356.5) in the study cohort compared to the control cohort, whereas an increase of 1.96% (886 to 869) was observed in the RNP (both periods *p* ≥ 0.05, Fig. 5; Table 4).

**Fig. 6:**
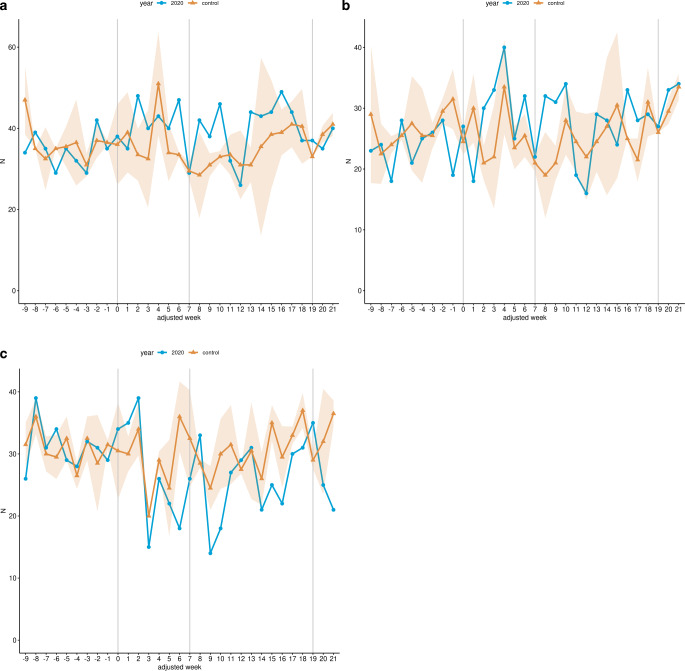
Hospital admissions for malignant neoplasm of head & neck stratified by treatment groups. Line charts of the cumulative weekly hospital admissions related to malignant neoplasms of head & neck across all 14 participating sites for the study cohort (*blue*) and the control cohort (*yellow*) from January 13 (adjusted week –9) to August 16 (adjusted week 21) 2020 stratified by therapy categories. **a** Hospital admissions with radiotherapeutic procedures, without surgery-related procedures. **b** Hospital admissions with radiotherapeutic procedures, without surgery-related procedures and presence of chemotherapy. **c** Hospital admissions with presence of surgery-related procedures. The *shaded area* represents the standard deviation of the weekly average of the years 2018 and 2019. Additionally, the data are shown without time resolution as boxplots in supplemental Figure S6

Among HNC treatment groups, admissions with radiotherapy but without surgery increased by 13.84% (802 to 704.5, *p* < 0.05) within in the LP in 2020 in comparison to the control cohort (Fig. 6a; Table 4). Admissions with radiotherapy in which chemotherapy procedures were present during the stay increased by 11.18% (557 to 501, *p* ≥ 0.05; Fig. 6b; Table 4). In contrast, in admissions in which surgery-related procedures were performed, a reduction of 11.28% (531 to 598.5, *p* ≥ 0.05) was observed in the same period (Fig. 6c; Table 4).

Within the RNP, radiotherapy admissions with an absence of surgery-related procedures during the stay increased by 14.83% (511 to 445, *p* < 0.05; Fig. 6a) and radiotherapeutic admissions with presence of chemotherapy increased by 9.66% (352 to 321, *p* ≥ 0.05; Fig. 6b; Table 4). In contrast, admissions with the presence of surgery-related procedures were reduced by 13.31% (342 to 394.5, *p* ≥ 0.05) in the same period compared with the control cohort (Fig. 6c; Table 4).

## Discussion

In this retrospective study, we analyzed changes in the therapeutic management of inpatients with CC and HNC across 14 German university hospitals following the lockdown announcement on March 16, 2020, in Germany [21]. A significant decrease in performed radiotherapy fractions for malignant neoplasms of the cervix uteri was observed across all participating sites in 2020 in the 20 weeks following the lockdown announcement on March 16, 2020, in Germany to August 2, 2020, compared to the average of the two previous years. This effect was especially driven by a reduction of Megavoltage radiation therapy (OPS 8‑522, 8‑523), whereas no differences were observed for brachytherapy (OPS 8‑524, 8‑525). Notably, even though on April 28, 2020, the German Federal Ministry of Health announced the gradual reactivation of hospital capacity for elective treatments from May onwards [20], the observed reduction was even more pronounced when analyzing the time period from May 4, 2020, to August 2, 2020, in more detail. These numbers are in accordance with our analysis of related hospital admissions.

The observed decrease in case numbers for CC with associated radiotherapy goes hand in hand with little, non-significant changes in hospitalizations treated surgically. In light of the restricted operating theater and intensive care capacity, the unlikely finding of a decrease is surprising but may be explained by a high priority of surgical cancer treatment at a time of reduced capacity for elective interventions.

The delayed decrease of radiotherapy-related hospitalizations might be a consequence of a curtailed oncological screening for CC relation to the pandemic. This reasoning would entail the presumption of a lag between the initial diagnosis and initiation of treatment of several weeks. As an alternative interpretation, these findings might be a consequence of intended treatment postponements. However, few radiation oncology institutes in Germany reported a postponement of treatment as a consequence of the pandemic [7]. From an outcome perspective, there exist no valid data on the effects of a delayed treatment in CC [24].

In contrast to other countries [25], there was no official suspension of CC screening during the corona lockdown in Germany. However, out of fear and because of the call to reduce contacts, women might have abstained from screening during this period. Such a reduced willingness might have detrimental effects on patients and treatment success [26]. This is in line with data based on German practices where, among other disciplines, gynecology practices showed a strong reduction in case numbers by 21.7 to 30.8% between March and May 2020, [27]. Another analysis based on the same data source as our study but addressing inpatient admissions in general, found that the decline in case numbers started immediately after introduction of the lockdown restrictions [13].

In contrast, for HNC, a significant increase in performed radiotherapeutic fractions was observed in our cohort in 2020 in the 20 weeks following the lockdown announcement on March 16, 2020, as well as in the 13-week period from May 4, 2020 onwards, in comparison with the average of the two previous years. In our analysis of related inpatient hospital admissions, an increase could be observed for admissions in which radiotherapeutic procedures were performed, whereas no differences could be observed for radiotherapeutic admissions with additional chemotherapy.

The increase in case numbers and fractions found in HNC was accompanied by a numerical decrease in cases with surgery, which was, however, not statistically significant. Here, respective changes in radiotherapy occurred after initiation of the lockdown measures with a delay of 1 to 2 weeks only. This delay is explainable by radiotherapy planning prior to hospitalization. The shift in hospitalized cases might reflect a preference for non-surgical treatments during the lockdown. Such a reasoning might especially apply to head and neck cancer, where surgery is complex and imposes significant COVID-related risks for the surgical team [3, 26]. One guideline recommended such a temporary shift from surgery to radiotherapy during the onset of the pandemic [1]. In contrast to our findings, Spencer et al. found no relevant change in the number of courses and attendances in their study for HNC in the already mentioned British data [5]. However, due to the centralized and unified character of the British health care system in the form of the NHS, measures and guidelines might have been introduced more coherently and stringently. In addition, the British study encompassed in- and outpatient data, with only a few centers failing to provide data.

Apart from this shift, other factors might have contributed to this finding: university hospitals with their large capacities might have received more patients in the aftermath of the first wave of the pandemic; diagnoses might be delayed, resulting in more advanced cases with different treatment approaches. In addition, the proportion of cases treated in an in- or outpatient setting might have changed. This applies to CC (outpatient treatment preferred during/after the lockdown) and HNC (inpatient treatment preferred during/after the lockdown). Finally, alternating chemotherapy regimens might have contributed to a change in admissions and fractions administered during hospitalization.

In a survey performed among radiation oncologists in Germany, Austria, and Switzerland, most of the radiation oncology institutes (ROIs) reported no change in curative or palliative treatment [7]. Fractionation schedules were changed in 25.7% (curative radiotherapy) and 42.1% (palliative radiotherapy) of the ROIs, while the general postponement of treatment played virtually no role. The authors also found that non-university ROIs were more willing to change their treatment pattern. This might well apply to our setting, which addressed only university institutions. The decrease in case numbers in the survey was independent of the regional incidence of COVID-19 and the type of institute (university vs. non-university).

### Limitations

The major limitation of the present analysis lies in the selective consideration of inpatients. However, radiotherapy might have shifted from an in- to an outpatient setting in the wake of the lockdown. We tried to mitigate this effect by focusing on entities with a strong inpatient component of treatment such as the regular use of concomitant radiochemotherapy. In addition, as the lockdown restrictions specifically targeted the inpatient setting while sparing outpatient cancer treatment and screening, a shift from in- to outpatient treatment would still in part be detectable in the hospital setting.

Regarding the observational unit of cases, confounded results might occur if shorter but repeated hospitalizations became the preferred pattern during the lockdown. However, such an alteration appears unlikely, as it would contradict the lockdown restrictions calling for reduced hospitalizations [19]. Thus, for assessment of temporal changes in radiotherapy, fractions are a more reliable endpoint than cases.

Furthermore, we have no detailed information on fractionation or dose concepts. In order to shorten treatments, hypofractionated or even ultra-hypofractionated radiotherapy might have become a frequently applied regime. An increased use of such ultra-hypofractionated concepts was especially striking in British data and the treatment of breast cancer [5]. The German Radiation Oncology Society (*Deutsche Gesellschaft für Radioonkologie*, DEGRO) recommended the application of hypofractionated concepts in order to reduce treatment time [28]. Two sources of alternated fractionation play an important role in HNC. On the one hand there might be a decrease in hyperfractionated concepts and an increase in the frequency of hypofractionation [18]. However, if such alterations were apparent in our data, we would underestimate the lockdown effect in terms of radiotherapy use, where we observed increased numbers during the lockdown period.

Admission varied considerably between institutions, decreasing the power to detect possible alterations caused by the lockdown. By applying mixed models, we could reduce the statistical variation between considered hospitals and estimate a generalized effect.

Another limitation may be the overlapping of cases between some therapy categories (Supplementary Table S1). For example, for CC, some cases of the category “radiotherapy without surgery, chemotherapy present” may also be included in the group “radiotherapy without surgery without brachytherapy.” However, we chose this approach to look at possible effects from different perspectives by analyzing the subgroups. Furthermore, the therapy category “surgery present” might include both “pure” surgical cases and those with additional radiochemotherapy during the same hospital stay. Although we assume that the latter is rather a minority, future analyses may aim at a stricter and more finely granulated separation between these therapy categories.

Further limitations introduced by the use of the claims dataset were also described in more detail in [13].

## Conclusion

The first COVID-19 lockdown had specific effects on the inpatient management of cervical and head and neck cancer in Germany. This led to increased admission of HNC but a delayed reduction in cervical cancer admissions. Future studies need to address potential effects on clinical outcomes.

## Supplementary Information

Figure S1: Radiotherapeutic fractions for malignant neoplasm of cervix uteri

Figure S2: Hospital admissions for malignant neoplasm of cervix uteri

Figure S3: Hospital admissions for malignant neoplasm of cervix uteri stratified by treatment groups

Figure S4: Radiotherapeutic fractions for malignant neoplasm of head & neck

Figure S5: Hospital admissions for malignant neoplasm of head & neck

Figure S6: Hospital admissions for malignant neoplasm of head & neck stratified by treatment groups

Table S1: Inclusion and Exclusion criteria
