# Supplementary figures and images for: Shift of radiotherapy use during the first wave of the COVID-19 pandemic? An analysis of German inpatient data
Source: Strahlenther Onkol. 2022 Jan 7;198(4):334–45. doi: 10.1007/s00066-021-01883-1 (PMC8739685; doi:10.1007/s00066-021-01883-1)

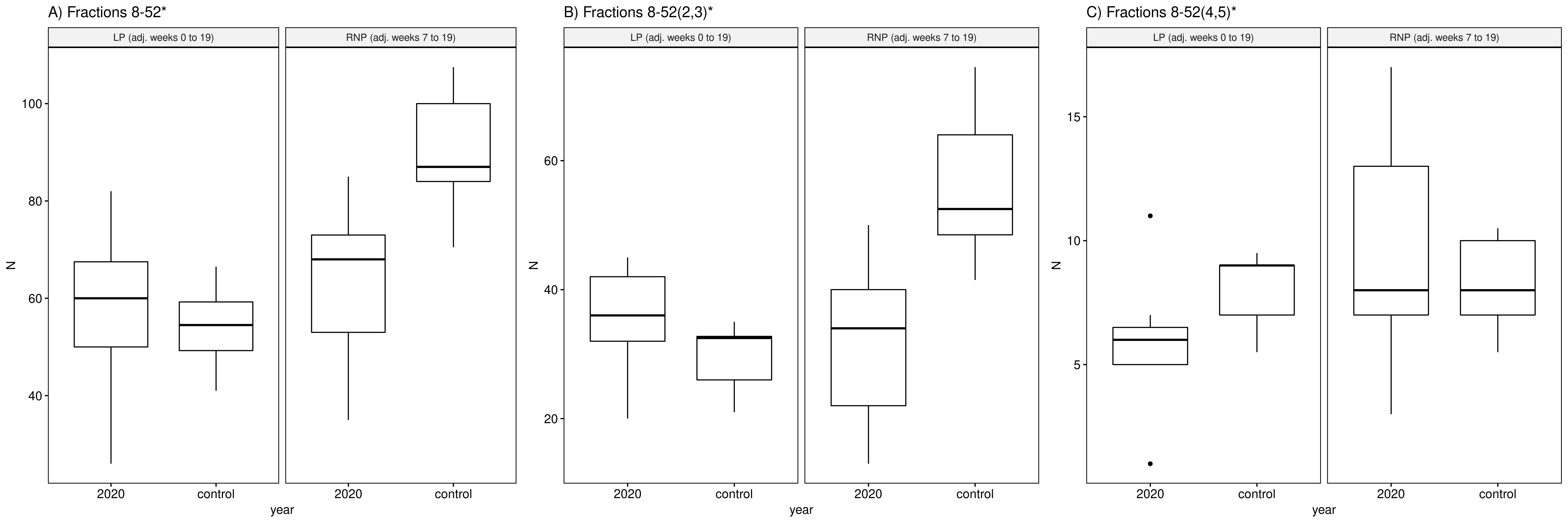

Supplement: Supplementary file 1 — Figure S1: Radiotherapeutic fractions for malignant neoplasm of cervix uteri [file 66_2021_1883_MOESM1_ESM.tiff]

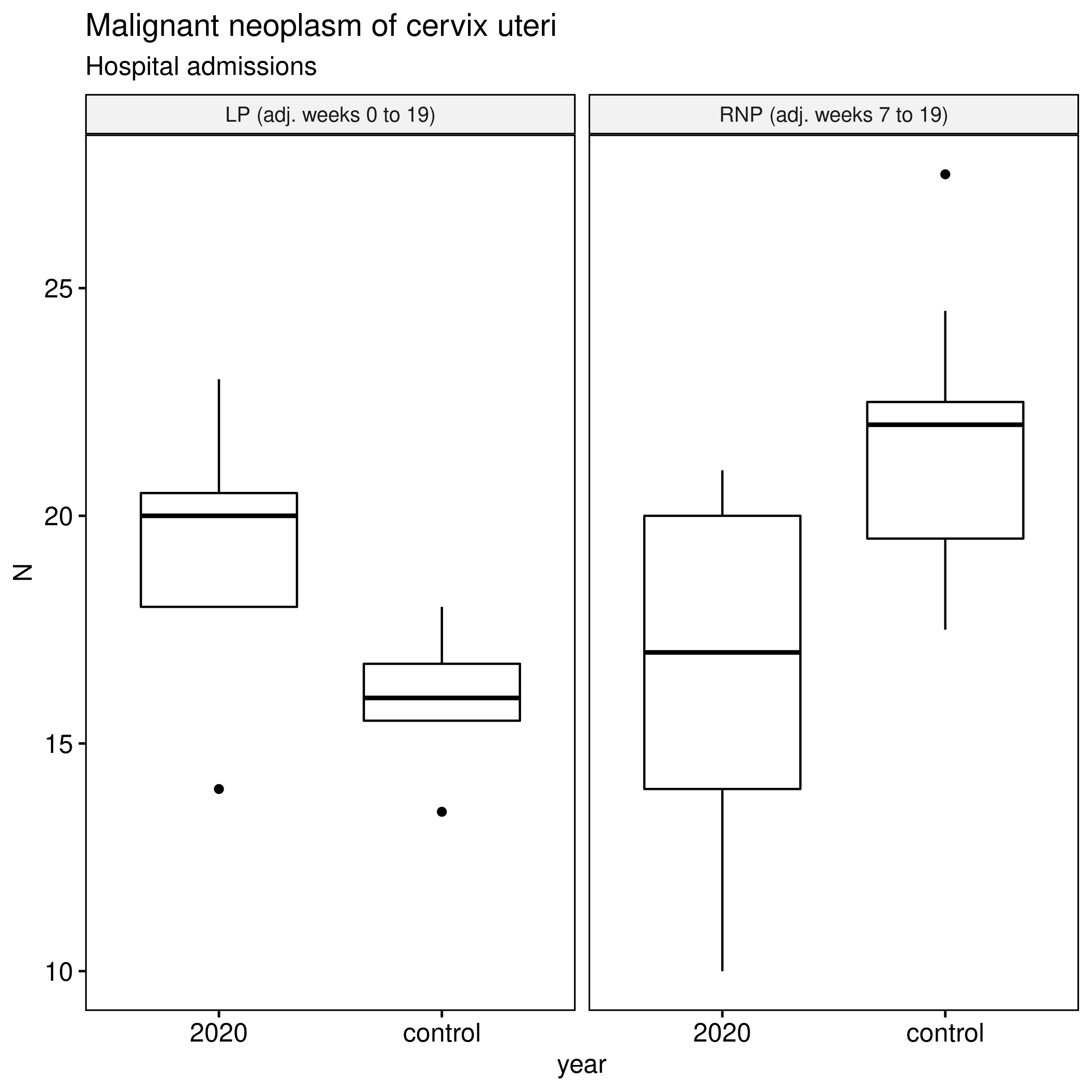

Supplement: Supplementary file 2 — Figure S2: Hospital admissions for malignant neoplasm of cervix uteri [file 66_2021_1883_MOESM2_ESM.tiff]

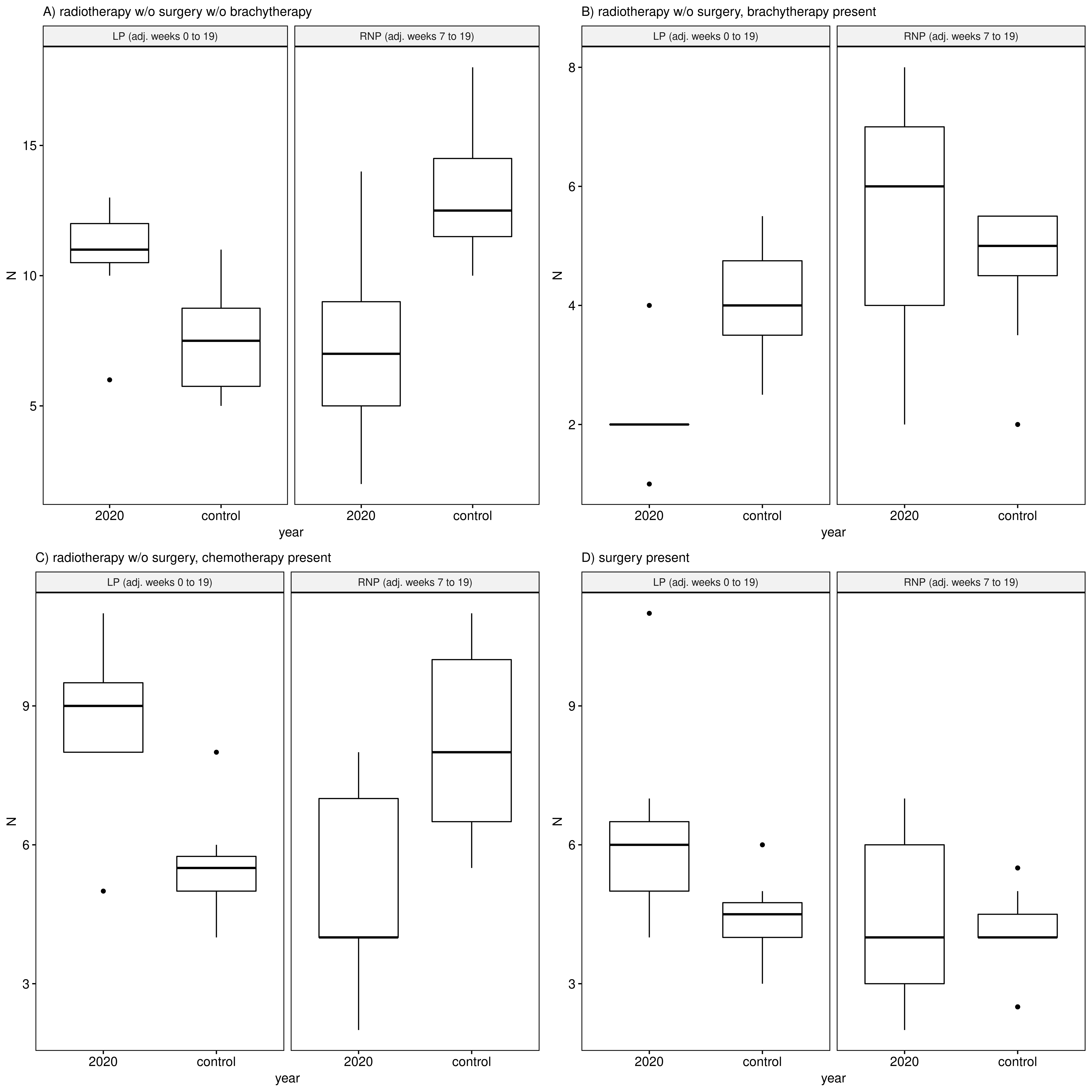

Supplement: Supplementary file 3 — Figure S3: Hospital admissions for malignant neoplasm of cervix uteri stratified by treatment groups [file 66_2021_1883_MOESM3_ESM.tiff]

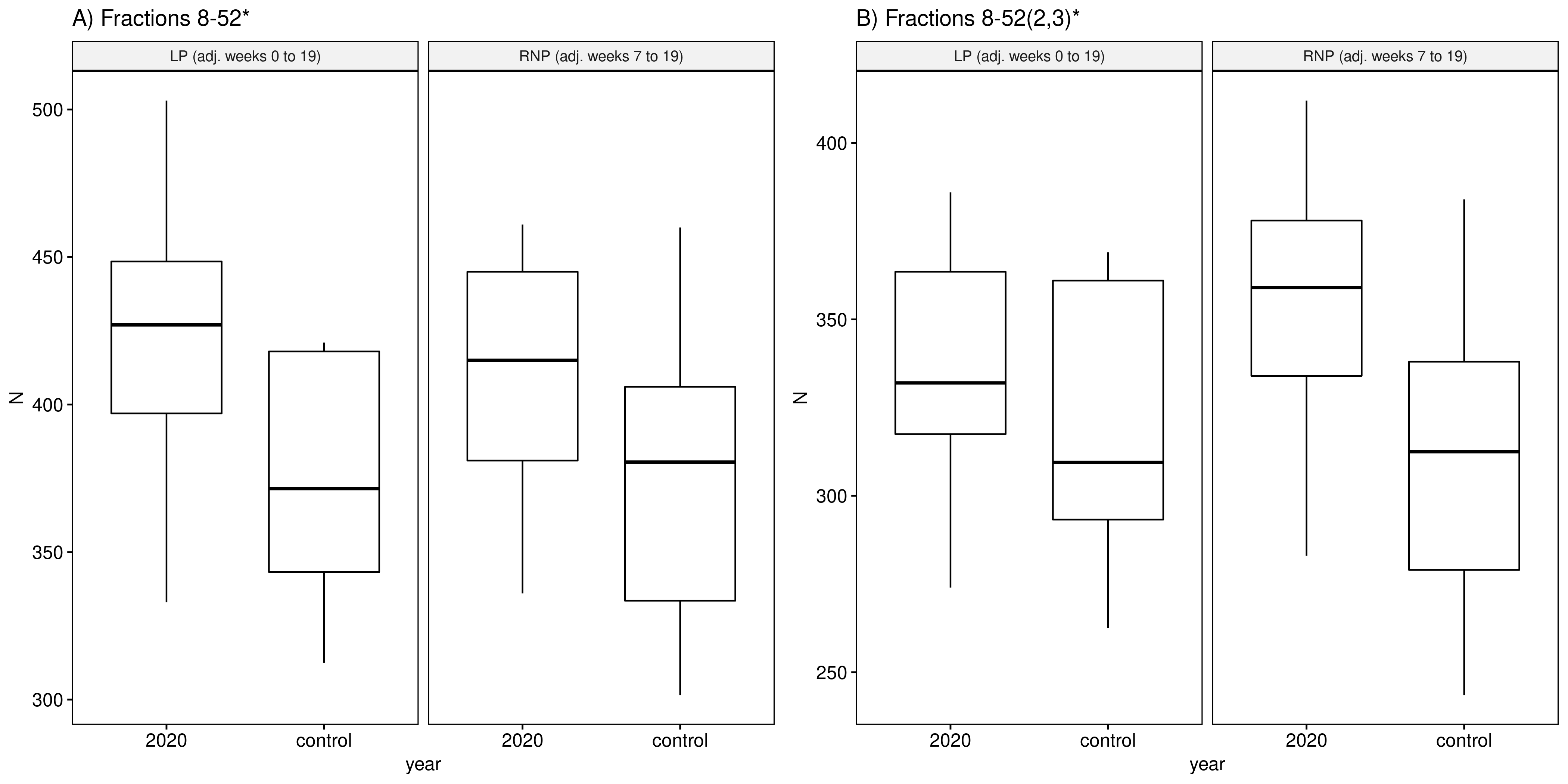

Supplement: Supplementary file 4 — Figure S4: Radiotherapeutic fractions for malignant neoplasm of head & neck [file 66_2021_1883_MOESM4_ESM.tiff]

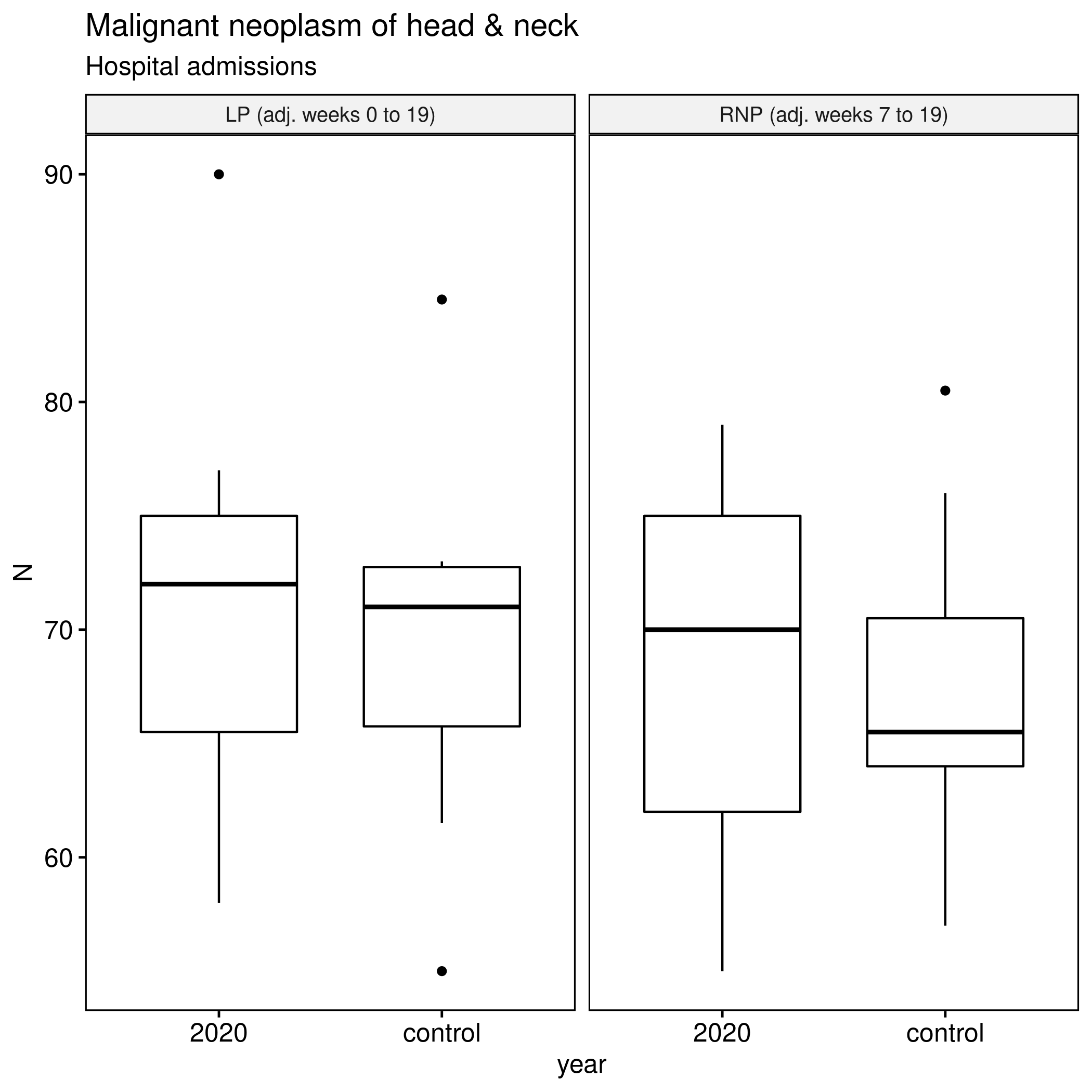

Supplement: Supplementary file 5 — Figure S5: Hospital admissions for malignant neoplasm of head & neck [file 66_2021_1883_MOESM5_ESM.tiff]

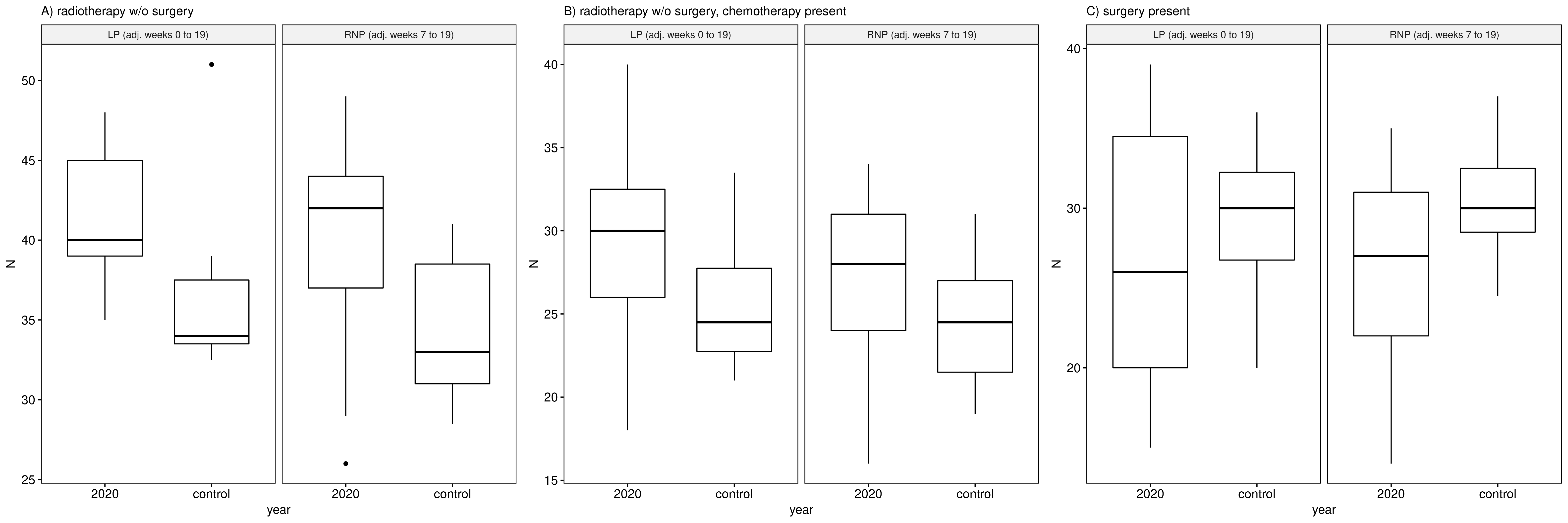

Supplement: Supplementary file 6 — Figure S6: Hospital admissions for malignant neoplasm of head & neck stratified by treatment groups [file 66_2021_1883_MOESM6_ESM.tiff]
