# Supplementary material for: Shift of radiotherapy use during the first wave of the COVID-19 pandemic? An analysis of German inpatient data
Source: Strahlenther Onkol. 2022 Jan 7;198(4):334–45. doi: 10.1007/s00066-021-01883-1 (PMC8739685; doi:10.1007/s00066-021-01883-1)
Supplement: Supplementary file 7 — Table S1: Inclusion and Exclusion criteria [file 66_2021_1883_MOESM7_ESM.docx]

#### Table S1: Inclusion and exclusion criteria.

| **Treatment group** | **Diagnosis group** | **Therapy category** | **Principal ICD codes (inclusion)** | **Secondary ICD codes (exlusion)** | **OPS codes (inclusion)** | **OPS codes 2 (inclusion)** | **OPS codes (exclusion)** |
| --- | --- | --- | --- | --- | --- | --- | --- |
| 1 | Malignant neoplasm of head & neck | surgery present | C00 | C78, C79 | 5-894.00, 5-894.04, 5-894.05, 5-894.10, 5-894.14, 5-894.15 |  |  |
|  |  |  | C01, C02 |  | 5-250.2, 5-251, 5-252 |  |  |
|  |  |  | C04, C05 |  | 5-272, 5-273, 5-277 |  |  |
|  |  |  | C07, C08 |  | 5-262 |  |  |
|  |  |  | C09, C10, C11, C13 |  | 5-290, 5-292, 5-295, 5-296 |  |  |
|  |  |  | C32 |  | 5-30 |  |  |
| 2 | Malignant neoplasm of cervix uteri |  | C53 |  | 5-681, 5-682, 5-683, 5-684, 5-685, 5-686, 5-687 |  |  |
| 3 | Malignant neoplasm of head & neck | radiotherapy w/o surgery | C00 |  | 8-522 |  | 5-894.00, 5-894.04, 5-894.05, 5-894.10, 5-894.14, 5-894.15 |
|  |  |  | C01, C02 |  |  |  | 5-250.2, 5-251, 5-252 |
|  |  |  | C07, C08 |  |  |  | 5-262 |
|  |  |  | C09, C10, C11, C13 |  |  |  | 5-290, 5-292, 5-295, 5-296 |
|  |  |  | C32 |  |  |  | 5-30 |
| 4 | Malignant neoplasm of head & neck | radiotherapy w/o surgery, chemotherapy present | C00 |  |  | 8-542, 8-543 | 5-894.00, 5-894.04, 5-894.05, 5-894.10, 5-894.14, 5-894.15 |
|  |  |  | C01, C02 |  |  |  | 5-250.2, 5-251, 5-252 |
|  |  |  | C04, C05 |  |  |  | 5-272, 5-273, 5-277 |
|  |  |  | C07, C08 |  |  |  | 5-262 |
|  |  |  | C09, C10, C11, C13 |  |  |  | 5-290, 5-292, 5-295, 5-296 |
|  |  |  | C32 |  |  |  | 5-30 |
| 5 | Malignant neoplasm of cervix uteri |  | C53 |  |  |  | 5-681, 5-682, 5-683, 5-684, 5-685, 5-686, 5-687 |
| 6 |  | radiotherapy w/o surgery, brachytherapy present |  |  | 8-524, 8-525.0, 8-525.1 |  |  |
| 7 |  | radiotherapy w/o surgery w/o brachytherapy |  |  | 8-522 |  | 5-681, 5-682, 5-683, 5-684, 5-685, 5-686, 5-687, 8-524, 8-525 |

Inclusion and exclusion criteria defined by a combination of primary and secondary ICD-10 diagnosis codes and OPS procedure codes. Each row of the table describes an individual combination of inclusion and exclusion criteria. The logical connection is as follows: logical "AND" between two inclusion criteria columns and logical "AND NOT" between inclusion and exclusion criteria columns. Multiple codes within one cell are interconnected with each other using a logical "OR". C00: Malignant neoplasm of lip. C01: Malignant neoplasm of base of tongue. C02: Malignant neoplasm of other and unspecified parts of tongue. C04: Malignant neoplasm of floor of mouth. C05: Malignant neoplasm of palate. C07: Malignant neoplasm of parotid gland. C08: Malignant neoplasm of other and unspecified major salivary glands. C09: Malignant neoplasm of tonsil. C10: Malignant neoplasm of oropharynx. C11: Malignant neoplasm of nasopharynx. C13: Malignant neoplasm of hypopharynx. C32: Malignant neoplasm of larynx. C53: Malignant neoplasm of cervix uteri.
